# Robust metrics for assessing the performance of different verbal autopsy cause assignment methods in validation studies

**DOI:** 10.1186/1478-7954-9-28

**Published:** 2011-08-04

**Authors:** Christopher JL Murray, Rafael Lozano, Abraham D Flaxman, Alireza Vahdatpour, Alan D Lopez

**Affiliations:** 1 Institute for Health Metrics and Evaluation, University of Washington, 2301 Fifth Ave., Suite 600, Seattle, WA 98121, USA; 2 University of Queensland, School of Population Health, Brisbane, Australia

**Keywords:** Verbal autopsy, metrics, validation

## Abstract

**Background:**

Verbal autopsy (VA) is an important method for obtaining cause of death information in settings without vital registration and medical certification of causes of death. An array of methods, including physician review and computer-automated methods, have been proposed and used. Choosing the best method for VA requires the appropriate metrics for assessing performance. Currently used metrics such as sensitivity, specificity, and cause-specific mortality fraction (CSMF) errors do not provide a robust basis for comparison.

**Methods:**

We use simple simulations of populations with three causes of death to demonstrate that most metrics used in VA validation studies are extremely sensitive to the CSMF composition of the test dataset. Simulations also demonstrate that an inferior method can appear to have better performance than an alternative due strictly to the CSMF composition of the test set.

**Results:**

VA methods need to be evaluated across a set of test datasets with widely varying CSMF compositions. We propose two metrics for assessing the performance of a proposed VA method. For assessing how well a method does at individual cause of death assignment, we recommend the average chance-corrected concordance across causes. This metric is insensitive to the CSMF composition of the test sets and corrects for the degree to which a method will get the cause correct due strictly to chance. For the evaluation of CSMF estimation, we propose CSMF accuracy. CSMF accuracy is defined as one minus the sum of all absolute CSMF errors across causes divided by the maximum total error. It is scaled from zero to one and can generalize a method's CSMF estimation capability regardless of the number of causes. Performance of a VA method for CSMF estimation by cause can be assessed by examining the relationship across test datasets between the estimated CSMF and the true CSMF.

**Conclusions:**

With an increasing range of VA methods available, it will be critical to objectively assess their performance in assigning cause of death. Chance-corrected concordance and CSMF accuracy assessed across a large number of test datasets with widely varying CSMF composition provide a robust strategy for this assessment.

## Background

Verbal autopsy (VA) has been in use in various field studies, surveillance sites, and national systems for more than four decades [1-4]. The instruments and analytical tools used to assign cause of death are rapidly evolving. New automated methods [4-7] have been proposed and are in use alongside traditional physician-certified verbal autopsy (PCVA). With new Bayesian statistical methods and machine learning approaches being developed, we can expect a wide range of new methods and refinements of existing methods in the coming years. It will become increasingly important for users of VA instruments and analytical tools to compare the performance of all the options in a balanced, objective fashion.

Large, but we argue inadequate validation datasets in which VA is compared to medical records have been collected and reported in the literature for China and Thailand [8,9]. The multisite Population Health Metrics Research Consortium has collected a very large validation dataset for neonates, children, and adults in Mexico, Tanzania, India, and the Philippines. These studies, as opposed to all previous efforts, provide the opportunity to compare VA results to gold standard cause of death assignment based on strict clinical diagnostic criteria [10]. All of these datasets provide rich empirical opportunities to assess the validity of existing and proposed VA methods. Robust comparison of performance requires standardization of the metrics used to assess the validity of VA and respect of some basic principles for the validation of empirically-derived approaches. Many metrics, including cause-specific sensitivity, specificity, concordance, absolute error in cause-specific mortality fractions (CSMFs), relative error in CSMFs, and Cohen's kappa have been reported in the literature [2,8,9,11-22]. The purpose of this paper is to identify and discuss the key issues that must be addressed to choose a set of metrics for VA validation studies and make recommendations based on this assessment for future reporting.

A wide array of different types of VA methods has been proposed. We can classify the various methods into four groups, based on the nature of the task that they attempt to perform: 1) individual death cause assignment to a single cause, which includes PCVA and variants of Symptom Pattern, Tariff, and machine learning [2,9,21,23-27]; 2) individual death cause assignment to multiple causes with probabilities across causes for each death summing to 100%; 3) direct estimation of CSMFs without assigning causes to individual deaths; and 4) combined methods that use both direct estimation of CSMFs and individual cause of death assignment so that the sum of the individual cause of death assignments equals the CSMFs from direct estimation. Proposed metrics need to be useful for comparing the performance of methods across this entire spectrum. Further, the metrics and validation study design needs to be able to help identify methods that are likely to perform better than others in many diverse settings with varying population CSMFs and cause lists.

Published studies on the validity of verbal autopsy have used a wide variety of measures, many of them coming from the literature on the evaluation of diagnostic tests. Authors have generally reported measures of the performance of a VA method for assigning causes to individual deaths such as sensitivity, specificity, concordance, and more recently, kappa [8,9,11,12,14,16-20]. In addition, they have used measures to assess how well a VA method estimates CSMFs, including the sum of the absolute values of CSMF errors, average CSMF error, and relative error in CSMFs [2,8,9,11,12,14-17,21,22].

There are many other measures proposed in the literature on nominal association such as phi, contingency coefficient, adjusted contingency coefficient, Tschuprow's T, Cramer's V, and Matthews correlation coefficient [28-32]. When applied to the comparison of true cause and predicted cause, these measures capture in a single quantity how often the true cause is predicted correctly as a complex function of misclassification of the true negatives. In VA, however, different uses, such as a research study or monitoring population health, imply different priorities on correct individual cause assignment or accurate CSMF prediction. For this reason, we do not believe that the measures of nominal association that produce a single measure reflecting both will be useful. We focus in this paper on separate measures of individual cause assignment and CSMF accuracy following the general VA tradition. This approach is also required because some of the proposed VA methods, such as the method of King and Lu [33], do not predict individual causes of death, only the CSMFs directly. In other words, metrics that require the full N by N matrix of true and predicted cause to be complete cannot be applied to some VA methods.

## Methods

### Many metrics are a function of the CSMF composition of a test dataset

We use a simple hypothetical case of a VA method to demonstrate why some currently-reported metrics may be difficult to interpret in a robust fashion. This illustration uses a hypothetical case of a population with three causes of death: A, B, and C. Imagine a VA method (by which we mean the combination of the instrument and the analytical tool applied to generate cause of death assignments), method 1, that produces a predicted cause for each death. Table 1 shows the probability that for a given true cause, method 1 will assign the death to one of the three possible causes. We can consider the matrix of these probabilities as the fundamental attribute of a VA assignment method. Given the matrix of these probabilities and the CSMF composition of a test dataset, we can easily compute the standard array of metrics, including sensitivity, specificity, concordance, absolute error in CSMFs, and relative error in the CSMFs.

**Table 1:** The hypothetical method 1 shows the probability of assigning a death from a true cause to each of the three possible causes; the hypothetical method 2 differs only in the higher probability of assigning deaths from cause A to cause A.

Method 1		Estimated		
		
		A	B	C
		
True	A	0.70	0.03	0.27
	
	B	0.04	0.60	0.36
	
	C	0.065	0.585	0.35
				
		
**Method 2**		**Estimated**		
		
		A	B	C
		
**True**	A	**0.80**	0.02	0.18
	
	B	0.04	**0.60**	0.36
	
	C	0.065	0.585	**0.35**

We have created 500 test datasets by randomly varying the cause composition of the test set (using random draws from an uninformative Dirichlet distribution). We use the Dirichlet distribution because it creates an even distribution across all possible combinations of causes that sum to 100%. By holding constant the probabilities of classification as a function of each true cause as shown in Table 1, we have quantified the range of each metric due purely to changes in the test set cause composition. Table 2 shows the mean, median, maximum, and minimum values of each metric across the randomly-varied cause compositions. Because we are holding constant the probability of correct and incorrect classification of each true cause, sensitivity for each cause in these simulations does not vary. But specificity for each cause, kappa, overall concordance, summed absolute CSMF error, and relative CSMF error vary widely. The ranges are large enough that one cannot meaningfully compare results of a method from one test dataset with results for another method in a different test dataset. We have demonstrated using a simple case how VA method performance can be affected by CSMF composition of the test set in principle; in multiple applications of this approach to different real VA methods [25-27,34-36] we have also found that this theoretical result holds true.

**Table 2:** Range of values for selected cause-specific and overall metrics of individual cause assignment and CSMF estimation for two different hypothetical VA assignment methods across 500 test datasets where the cause composition of the test datasets has been randomly varied.

	Method 1				Method 2			
**Cause A**	**Mean**	**Median**	**Max**	**Min**	**Mean**	**Median**	**Max**	**Min**

**Sensitivity**	0.70	0.70	0.70	0.70	0.80	0.80	0.80	0.80

**Specificity**	0.95	0.95	0.96	0.94	0.95	0.95	0.96	0.94

**Absolute CSMF error**	0.08	0.06	0.29	0.00	0.05	0.04	0.19	0.00

**Relative CSMF error**	0.74	0.24	53.38	0.00	0.71	0.15	53.48	0.00

**Chance-corrected concordance**	0.55	0.55	0.55	0.55	0.70	0.70	0.70	0.70

**Estimated versus true regression**	**Intercept**	**Slope**		**RMSE**	**Intercept**	**Slope**		**RMSE**

	0.52	0.64		0.00	0.52	0.74		.00

								

**Cause B**	**Mean**	**Median**	**Max**	**Min**	**Mean**	**Median**	**Max**	**Min**

**Sensitivity**	0.60	0.60	0.60	0.60	0.60	0.60	0.60	0.60

**Specificity**	0.69	0.69	0.97	0.42	0.70	0.70	0.98	0.42

**Absolute CSMF error**	0.17	0.15	0.57	0.00	0.17	0.15	0.57	0.00

**Relative CSMF error**	4.50	0.37	229.07	0.00	4.43	0.37	228.56	0.00

**Chance-corrected concordance**	0.40	0.40	0.40	0.40	0.40	0.40	0.40	0.40

**Estimated versus true regression**	**Intercept**	**Slope**		**RMSE**	**Intercept**	**Slope**		**RMSE**

	0.30	0.29		0.11	0.30	0.30		0.11

								

**Cause C**	**Mean**	**Median**	**Max**	**Min**	**Mean**	**Median**	**Max**	**Min**

**Sensitivity**	0.35	0.35	0.35	0.35	0.35	0.35	0.35	0.35

**Specificity**	0.69	0.69	0.73	0.64	0.73	0.73	0.82	0.64

**Absolute CSMF error**	0.20	0.19	0.63	0.00	0.19	0.17	0.63	0.00

**Relative CSMF error**	6.75	0.50	793.85	0.00	6.01	0.49	780.54	0.00

**Chance-corrected concordance**	0.03	0.03	0.03	0.02	0.03	0.03	0.03	0.02

**Estimated versus true regression**	**Intercept**	**Slope**		**RMSE**	**Intercept**	**Slope**		**RMSE**

	0.31	0.03		0.01	0.26	0.08		0.03

								

**Overall causes**	**Mean**	**Median**	**Max**	**Min**	**Mean**	**Median**	**Max**	**Min**

**Kappa**	0.26	0.28	0.47	0.00	0.30	0.33	0.53	0.00

**Total absolute CSMF error**	0.46	0.45	1.26	0.01	0.42	0.37	1.26	0.03

**CSMF accuracy**	0.75	0.75	1.00	0.37	0.77	0.80	0.98	0.36

**Figure 1:**
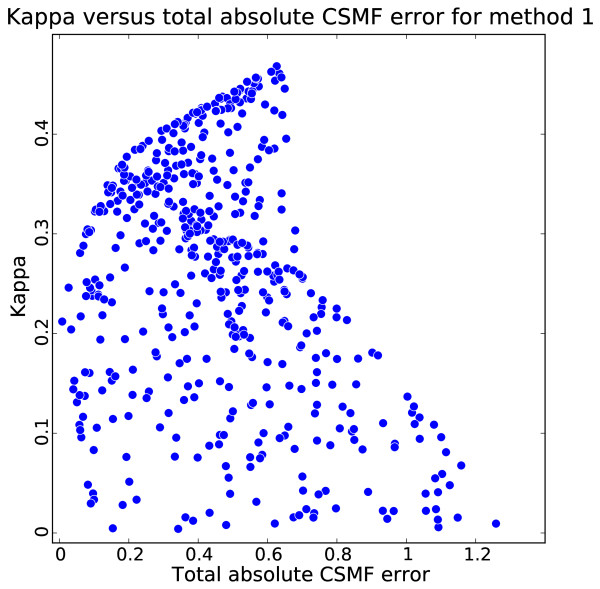
**Kappa versus total absolute CSMF error for method 1 for 500 iterations of experiment with varying true CSMFs**. This graph shows why kappa should not be used as a metric for CSMF accuracy.

Figure 1 compares a measure of performance for assigning cause to individual deaths, kappa, with the total absolute error in the CSMFs. This comparison highlights that a method's ability to assign individual causes is not closely related to how well it can estimate CSMFs. The reason is simple: even when sensitivities for the three causes are low and therefore kappa is low, false positives can be balanced by true negatives for each cause. When false positives and true negatives are exactly balanced, there will be no error in the estimated CSMFs. However, these simulations highlight that this can occur because of the particular and, quite possibly, idiosyncratic CSMF composition of the test dataset.

Even though results of all standard metrics except sensitivity are strongly affected by the CSMF composition of the test dataset, are comparisons of two VA methods made on one test dataset with one particular CSMF composition still robust? We can adapt this simple three-cause simulation environment to explore this question. Table 1 shows the probabilities of assigning each true cause to the three predicted causes for a second VA method, method 2. This method is superior to method 1. For true causes B and C it assigns the deaths in exactly the same proportions as method 1, but for cause A, sensitivity is higher in method 2, and the relative pattern of misclassification is the same. Using the same 500 test datasets with widely varying CSMF compositions, Table 3 counts the number of times that method 1 or 2 has better performance for absolute CSMF error by cause. In fact, 32%, 36%, and 49% of the time for cause A, cause B, and cause C, respectively, the inferior method (method 1) reports smaller absolute CSMF error. This simple finding illustrates how it could be extremely misleading to draw conclusions about the performance of one method compared to another on the basis of only one test dataset.

**Table 3:** The number of times method 1 or 2 has better performance for the absolute CSMF error in 500 randomly-generated test datasets with varying CSMF composition.

Cause	A		B		C	
**Method**	1	2	1	2	1	2

**Absolute CSMF error**	160	340	181	319	247	253

In any real comparison of alternative VA methods with longer cause lists, it is highly likely that for some causes, sensitivities will be higher and for others, lower. The pattern of misclassification is also likely to vary substantially. In these more complicated cases, drawing conclusions about which method performs better cannot be made based on one test dataset but needs to be carefully assessed for a diverse range of cause compositions in a series of test datasets.

These three-cause cases also point out that the performance of individual cause assignment in predicting the true cause correctly is quite distinct from how well a VA method does at predicting the true CSMFs. Clearly, when sensitivities for each cause equal 100% for all causes, the CSMFs will be correctly predicted. But for all realistic cases of VA where sensitivities will be far below 100%, we need to quantify the performance of a VA method both at assigning individual causes correctly and for predicting CSMFs accurately.

We explore metrics for individual cause assignment in more detail. The key issues examined include correcting for chance, dealing with the cause composition of the test dataset, and partial cause assignment metrics. In the following section, we discuss measures of CSMF accuracy, including the choice between measures of absolute and relative error, adjusting for the number of causes, comparison to random assignment and taking into account cause composition of the test set.

## Results

### Metrics for individual cause assignment

The performance assessment of a method that operates at the individual level has two components: the fraction of true deaths from a cause that are correctly assigned to that cause and the balance between true negatives (true deaths from that cause assigned to other causes) and false positives (deaths from other causes assigned to that cause). The balance between true negatives and false positives only matters as it affects the estimates of the CSMF. Given that we will recommend separate metrics for the accuracy of CSMF prediction, the only aspect of individual cause assignment that matters is whether the true cause is correctly predicted. In Table 1, these are the deaths in the diagonal cells of the matrix compared to the total number of deaths in each row. In the literature on diagnostic tests, the number of deaths in the diagonal cell divided by the total of the row is defined as the sensitivity for a given cause. The generalized version for multiple causes has been referred to as concordance [21,37,38]. As a measure of agreement for a cause, neither sensitivity nor concordance takes into account agreement expected by chance alone.

If we had a VA algorithm that randomly assigned deaths to each cause, we would expect it to have a concordance of (1/n), where n is the number of causes, as long as there are large numbers for each cause. In other words, if there are five causes of death and we randomly assign deaths to each of the five causes, we would be right 20% of the time. The general concept of correcting for concordance based on chance can be represented as:

Where the P(observed)*_j _*is the fraction that are correctly assigned for a cause j and P(expected)*_j _*is the fraction correctly assigned on the basis of chance alone. There are two choices that affect the exact formulation of this class of measures: whether to compute an overall measure of chance-corrected association and/or a cause-specific measure of chance-corrected association and how to estimate the association expected on the basis of chance alone.

There are at least two methods for estimating the P(expected).

1. Cohen's kappa calculated P(expected) as:

Where *p_ij _*is the probability of assigning a death of cause i to cause j. In addition, P(observed) is calculated as:

Note that since P(expected) and P(observed) are defined over all causes, Cohen's kappa is an overall-causes measure of chance-corrected association.

2. Cohen's kappa assumes that the chance prediction is informed by the true test set cause composition. A more naïve assumption, perhaps more appropriate for VA validation studies, is that the method is uninformed about the true test composition, and chance assignment would simply be equal assignment to all causes. An alternative method for estimating P(expected) is to assume it is simply (1/n), where n is the number of causes.

Cohen's kappa has been reported in the VA literature, but it is not the most attractive approach to correcting for chance in VA applications. As shown in Table 2, Cohen's kappa is quite sensitive to the cause composition of the test dataset, while option two above is not at all sensitive to this cause composition. Furthermore, Cohen's kappa provides a measure of association across all causes and not a cause-specific measure of concordance, although logically this approach to correcting for chance could be applied at the cause level.

Based on simplicity and the robustness to the CSMF composition of the test dataset, we propose to measure chance-corrected concordance for cause j (CCC_j_) as:

Where TP is true positives, TN is true negatives, and N is the number of causes. TP plus TN equals the true number of deaths from cause j.

Reporting this measure enhances the comparability across studies with different numbers of causes. When there are only a small number of causes, the chance-corrected concordance will be substantially lower than sensitivity. When a VA algorithm gets less than (1/n) fraction of the deaths correct for a cause, it will have a chance-corrected concordance that is negative. In all other cases, the chance-corrected concordance will range from 0 to 1.

In addition to reporting the chance-corrected concordance for each cause, we will also be concerned with how well a VA method performs overall at individual cause assignment for most applications of VA. This summary judgment requires a summary metric for VA individual cause assignment for a given test dataset of the form:

The question is how to choose the set of weights across causes to yield an overall summary for a given test dataset. There are three logical options available: the CSMFs in the test dataset, a standardized distribution of CSMFs such as the global cause of death distribution, and equal weights. Using the test set CSMFs appear to be undesirable, as the results across VA validation studies would not be comparable. If there is a positive or negative correlation between the chance-corrected concordances by cause and the CSMFs in the test set, the overall chance-corrected concordance will vary substantially. The second option, using weights equal to the global cause of death distribution as currently known, is appealing. The problem, however, is that in many validation studies, not all causes present in the global distribution are included. This can be handled as long as the validation study includes categories for other causes. But in a validation study on three or four specific causes with residual causes grouped under "other causes," the chance-corrected concordance for "other causes" would dominate the results if these were standardized to the global cause of death distribution. An alternative would be to rescale the cause fractions in the global distribution for each study such that the sum of the weights on the included causes equals one. But this would remove some of the appeal of using the global CSMFs as weights. The third option, in which the weights on each cause are equal for all causes included in the study, is the easiest to implement and the most comparable. Based on considerations of simplicity of explanation, ease of implementation, and comparability, we recommend the overall chance-corrected concordance be calculated as the average of the cause-specific chance-corrected concordances, namely equal weights, in the above equation.

Even when the overall chance-corrected concordance is calculated as the average of the cause-specific chance-corrected concordances, the CSMF composition of the test set may influence the result. Some more complex VA analytical methods may not have constant probabilities of assignment to causes conditional on the true cause of death. In other words, it is possible that concordance for a cause may vary as a function of the test dataset CSMFs. To avoid making the wrong inference on a method's performance, we recommend that a set of 100 or more test datasets be created with varying CSMF compositions using sampling with replacement of the test deaths by cause. Draws should be taken from an uninformative Dirichlet distribution to capture the range of possible CSMF compositions and sampling with replacement used to generate a range of test datasets. For each test dataset, the overall chance-corrected concordance should be estimated and the median value of these results should be reported as the single summary measure of individual cause assignment.

Some VA methods proposed or under development assign probabilities to more than one cause for each death [33,37]. These probabilities are assigned such that they sum to one for each death. There is literature on a range of measures for these types of cases [39,40]. These take into account the probability attached to the correct cause, not just its presence in the top k causes. For simplicity and ease of communication, we can compute a partial death assignment concordance as the fraction of deaths for which the true cause is included in the top k causes, ranked by their predicted probability. For example, a method could predict for a particular death that it is 50% tuberculosis, 20% pneumonia, 10% lung cancer, 10% AIDS, 5% heart failure, and 5% other infectious diseases. We can compute the fraction of the time that the true cause is the top cause (tuberculosis), the top two causes (tuberculosis or pneumonia), the top three causes, and so on. By definition, as the number of causes that are considered for calculating concordance (top two, top three, top four, etc.) increases, the calculated concordance must increase or at least remain equal.

As for single cause concordance, we should correct the partial cause concordance for how much better the VA method is than random assignment. The formula for the partial concordance from random assignment takes into account the combinatorics of cases where the same cause is selected at random more than once and simplifies to:

Where PC(k) is the partial concordance due to random assignment for the top k causes, and N is the number of causes in the study.

The partial chance-corrected concordance for the top k causes, PCCC(k) becomes:

Where C is the fraction of deaths where the true cause is in the top k causes assigned to that death. As k increases, it is not necessary that PCCC(k) increases. In fact, at the limit where k equals N, the PC(k) will equal 1.0, and the PCCC(k) will not be defined. By computing the PCCC(k), we facilitate comparisons across studies with different numbers of causes and perhaps different choices of k. As for individual cause assignment, median PCCC(k) across 100 or more test datasets in which the CSMFs have been sampled from an uninformative Dirichlet distribution should be reported.

### CSMF accuracy

When true negatives for a cause do not equal the false positives estimated for that same cause, the predicted CSMF will be too large or too small. A key choice in the design of metrics for CSMF accuracy is whether we are interested in absolute or relative errors in the CSMF. If the true CSMF for a cause is 15% and we predict 16%, this an error of one percentage point. If, for another cause, the true CSMF is 1% and we predict 2%, the error is also one percentage point. Should we be equally concerned about both of these one percentage point errors? Or is a doubling of the second cause from 1% to 2% a worse error than the 6.7% overestimation of the cause fraction for the first cause? This is the classic problem that has been discussed in several fields: whether we care about absolute or relative errors [41,42]. The answer is strictly a normative choice; as such, our answer must depend on how we intend to use VA results and what the consequences are of making various types of errors.

What are the potential effects of misclassification when true negatives do not equal false positives on population health or well-being? If the size of the burden of a problem influences the allocation of resources to programs or research or changes the allocation of managerial or political attention, then inaccurate CSMFs could affect health or well-being. In this sense, is the harm from inaccurate CSMFs related to absolute or relative errors? Financial resources will have less health impact if we move resources away from cost-effective intervention areas to less cost-effective areas. Such harm would be related to the absolute error in the CSMF, not the relative error. Imagine a case where we underestimate the CSMF by 100 deaths for a cause of death with a highly cost-effective intervention strategy available. Because we have underestimated the magnitude of the cause, fewer resources are allocated to the program dealing with this cause, and resources are moved to address a health problem that has been overestimated but for which the intervention strategy is less cost-effective. The misallocation of resources translates in this hypothetical case into 10 fewer lives being saved. The reduction in the number of lives saved is a negative consequence that can be traced to the misestimation of the CSMFs. Resources scale to the absolute size of problem (and cost effectiveness of interventions). In this example, which can be confirmed in an optimization model, the negative consequence scales to the absolute error in cause estimation, not the relative error. In the absence of a detailed understanding of which causes have more or less cost-effective intervention strategies and how over- or underestimation will lead to misallocation of resources, it appears prudent to treat all deaths misclassified where true negatives and false positives are not in balance as equally problematic. In other words, we should be concerned with absolute errors in the CSMFs, not relative errors. Given that negative consequences can come from underestimation or overestimation, we should, in fact, be interested in the absolute value of absolute errors in the CSMFs across each cause. For a summary metric across all causes, we could report the average of the absolute value of the CSMF error.

Absolute errors in the CSMFs will tend to be smaller the larger the number of causes in the cause list. For any given cause list, the maximum possible average or total error would occur when we estimate 100% of all deaths due to the cause with the smallest true cause fraction. For any given number of causes, the total of the absolute value of the CSMF errors across causes will always be

The average of the absolute value of the errors is this quantity divided by N, where N is the number of causes. This convenient result means that we can compute the performance of any VA method compared to the worst possible method. This comparison is then independent of the number of causes in the cause list. Therefore, we define CSMF accuracy as:

This quantity will always range from zero to one, where a value of one means no error in the predicted CSMFs and a value of zero means the method is equivalent to the worst possible method of assigning cause fractions.

Cause composition of the test set can matter because chance assignment does better or worse depending on the test set. Perhaps more important are two other reasons that CSMF composition can influence the results. First, as shown in Table 2, even when the percentage distribution of a true cause is constant across predicted causes - for example, for true cause A, 50% are assigned to A, 30% to B, and 20% to C - variation in true CSMFs changes the CSMF average absolute error dramatically. Second, for some of the more complex VA methods, the probability of the predicted cause conditional on the true cause will also vary as a function of the cause composition of the test set. Since the purpose of VA validation studies is to identify which method will work in a variety of population epidemiological conditions, reporting CSMF error or CSMF accuracy for one test set would risk drawing an incorrect inference on relative performance.

Given that the CSMF composition of the test set can have multiple influences, to generate robust conclusions about the performance of one VA method compared to another, the cause composition of the test set should be varied using resampling methods. We can use draws from an uninformative Dirichlet distribution to evenly sample all possible cause compositions that sum to one. The Dirichlet distribution can be used because we can generate widely varying cause compositions of the test dataset that sum to 100% for any number of causes. Further, the expected value for each cause of the uninformative Dirichlet is equal cause fractions, but for any given draw from the distribution there is a wide range of cause fractions. For each sample from the cause composition, we can sample the test data with replacement to generate a new matching dataset with an alternative cause composition. After generating predictions for each alternative test dataset using a proposed VA method, we can compute CSMF accuracy. A summary metric would be the median CSMF accuracy across the draws. The median value will be the preferred metric in this case because CSMF accuracy can take on extreme values for some cause compositions.

Repeated draws from the uninformative Dirichlet distribution should be continued until the median value of CSMF accuracy stabilizes. Graphing the median value as a function of the number of draws can provide a visual indication of at what point CSMF accuracy changes little with further sampling. The number of draws depends on the tolerance for changes in the median. A reasonable tolerance is that further draws do not alter the median value by more than 0.5%.

**Figure 2:**
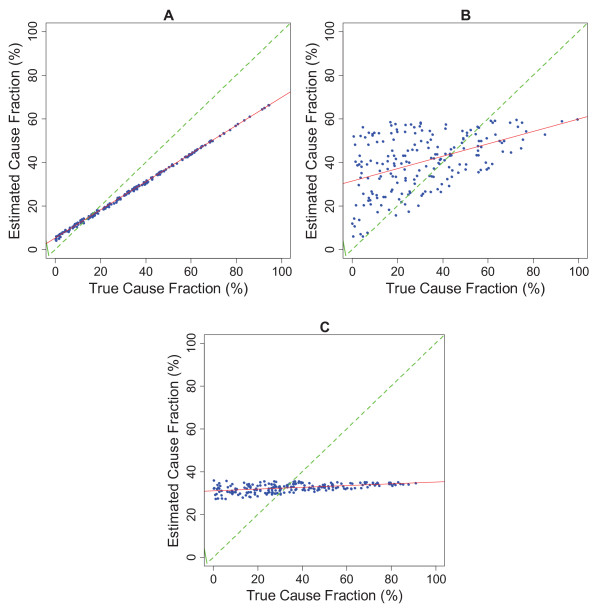
**Estimated CSMF versus true CSMF for causes A, B, and C using method 1 for 500 iterations of experiment with varying true CSMFs**.

Many users of verbal autopsy will also be interested in the robustness of CSMF estimation for specific causes. CSMF performance can be assessed by examining the relationship between the estimated CSMF for a cause and the true CSMF for a cause. Because several hundred test datasets have been created by sampling from an uninformative Dirichlet distribution and then sampling with replacement from the test data, it is possible to examine the relationship between estimated CSMF and true CSMF cause by cause. Figure 2 illustrates the relationship between estimated and true CSMFs using the hypothetical VA method 1 across the 500 test datasets for causes A, B, and C. There are three important aspects that relate to CSMF performance that can be best understood in terms of the relationship between the estimated CSMF and the true CSMF:

The intercept in the relationship between estimated CSMF and true CSMF, *α*, is an indication of how much a method tends to assign deaths to a cause even when there are no deaths from that cause in the test dataset. Some methods tend towards assigning an equal share of deaths to each cause. These methods will tend to have large nonzero intercepts that approach in the extreme (1/*n*), where *n *is the number of causes. The slope of the relationship, *β*, indicates by how much the estimated CSMF increases for each one percentage point in the true CSMF. Because some or many causes are nonzero intercepts, the slopes for almost all causes for almost all methods will be below 1. In other words, most methods will tend to overestimate small causes and underestimate large causes. The slopes, however, will be highly variable. Finally, the error term in the relationship between estimated and true CSMF provides an indication of how much an estimated cause fraction varies given a particular value of the true cause fraction. Using Ordinary Least Squares regression, the values for α, β, and the standard deviation of the error term (root mean squared error [RMSE]) can be estimated and reported by cause. These three values provide an easily-interpreted assessment of the performance of a VA method at estimating the CSMF for a given cause.

## Discussion

Our explication of performance metrics for VA leads to the following conclusions. First, for VA methods that assign individual causes to deaths, chance-corrected concordance should be reported for each cause, and the average chance-corrected concordance should be used as a summary measure of individual cause assignment. Second, for VA methods that assign multiple causes to deaths, the partial chance-corrected concordance for the top k causes should be reported for each cause, and the average partial chance-corrected concordance for the top k causes should be used as a summary measure. Third, for all VA methods, median CSMF accuracy computed for a set of test datasets with different CSMF composition drawn from an uninformative Dirichlet distribution should be reported.

Because some readers of VA validation studies may not want a single summary measure of performance for assigning individual causes of death or a single summary of CSMF estimation, it will be important to make available the full N by N classification matrix comparing true to assigned cause for all the test datasets. While for most readers this detail will be hard to interpret, it is an important aspect of transparency for validation studies to have this information available at least on demand.

For methods that are based on empirical patterns in the data, such as machine learning, Symptom Pattern, Tariff, direct CSMF estimation, or combined methods, great care needs to be taken to ensure that the data used to test the validity of the proposed method are not used for developing or "training" the method. These methods are extremely effective at identifying patterns in the data and can easily overfit the data. Strict separation of the test and training data is a critical aspect of any validation study. To avoid chance results from a particular train-test split in the data, validation studies for empirical methods should use multiple train-test splits and report the distribution of values for chance-corrected concordance and median CSMF accuracy. It is also essential to ensure that the CSMF composition of the test datasets is selected at random and is not the same as the CSMF composition of the training datasets. To simplify computational needs, the steps of generating different train-test splits and varying the CSMF composition of the test data through resampling can be combined.

Several published studies [43,44] have used Cohen's kappa as a measure of how accurately CSMFs are predicted by the method. In fact, Cohen's kappa is a summary measure of how well individual causes of death are assigned. CSMF errors of near zero are possible with kappa values that are less than 0.1. Cohen's kappa is an alternative to average chance-corrected concordance; it is not a measure of CSMF estimation error. Cohen's kappa, however, will be influenced by the composition of the test training set, as illustrated in Table 2, while average chance-corrected concordance is not affected by the test set cause composition.

## Conclusion

Even if other measures are reported in addition to those recommended here, inclusion of this standard set of metrics will facilitate comparison across different studies with likely different numbers of causes and different CSMF compositions. The metrics reported here will also encourage an explicit recognition of the potential tradeoffs for some methods between individual cause assignment and CSMF accuracy. Different users are likely to attach different importance to these dimensions; making standardized measurements of both dimensions available for all VA methods will facilitate choosing among the different options. These two standard metrics also reflect the principal information needs of the main users of cause of death data, namely population-level monitoring of leading causes of death (policy) and risk attribution in epidemiological enquiries (research). We expect that standardized metrics will facilitate further methods innovation in the future by providing a clear answer if a new method is leading to improved performance either in the dimension of individual cause assignment or CSMF accuracy. Future validation studies of verbal autopsy methods will also have greater credibility, not only if the appropriate metrics are used, but also if great care is taken in establishing true gold standard cause of death assignment. In the absence of rigorous gold standards, reporting chance-corrected concordance and CSMF accuracy will remain only measures of similarity between two imperfect assessments of cause of death. Robust validation studies require the right metrics as well as the appropriate study design.

## Abbreviations

CSMF: cause-specific mortality fraction; PCCC: partial chance-corrected concordance; PCVA: physician-certified verbal autopsy; RMSE: root mean squared error; VA: verbal autopsy

## Competing interests

The authors declare that they have no competing interests.

## Authors' contributions

CJLM, RL, and ADL conceptualized the study and guided analyses. ADF and AV performed analyses and helped write the manuscript. CJLM drafted the manuscript and approved the final version. CJLM accepts full responsibility for the work and the conduct of the study, had access to the data, and controlled the decision to publish. All authors have read and approved the final manuscript.
