# Correction: Robust metrics for assessing the performance of different verbal autopsy cause assignment methods in validation studies

**DOI:** 10.1186/1478-7954-12-7

**Published:** 2014-04-10

**Authors:** Christopher JL Murray, Rafael Lozano, Abraham D Flaxman, Alireza Vahdatpour, Alan D Lopez

**Affiliations:** 1 Institute for Health Metrics and Evaluation, University of Washington, 2301 Fifth Ave., Suite 600 Seattle, WA 98121, USA

## Correction

After publication of this manuscript [1], it came to the attention of the authors that the manuscript contained an error in the equation and text on page 5. The corrected version is below.

Based on simplicity and the robustness to the CSMF composition of the test dataset, we propose to measure chance-corrected concordance for cause j (CCC_j_) as:

$$\mathrm{CC}C_{j}=\frac{\left(\frac{TP_{j}}{TP_{j}+FN_{j}}\right)-\left(\frac{1}{N}\right)}{1-\left(\frac{1}{N}\right)}$$

Where TP is true positives, FN is false negatives, and N is the number of causes, TP plus FN equals the true number of deaths from cause j.

The Publisher and authors apologize to the readers for any inconvenience caused.
